# Association between Dietary Patterns and Serum Hepatic Enzyme Levels in Adults with Dyslipidemia and Impaired Fasting Plasma Glucose

**DOI:** 10.3390/nu13030987

**Published:** 2021-03-18

**Authors:** Li-Yin Lin, Chien-Yeh Hsu, Hung-Yi Chiou, Hsiu-An Lee, Li-Ming Hsu, Po-Ya Chang, Adi Lukas Kurniawan, Jane C.-J. Chao

**Affiliations:** 1 School of Nutrition and Health Sciences, College of Nutrition, Taipei Medical University, 250 Wu-Hsing Street, Taipei 11031, Taiwan; jlin11025@gmail.com; 2 Master Program in Applied Epidemiology, College of Public Health, Taipei Medical University, 250 Wu-Hsing Street, Taipei 11031, Taiwan; hychiou@tmu.edu.tw; 3 Institute of Population Health Sciences, National Health Research Institutes, 35 Keyan Road, Zhunan Town, Miaoli County 35053, Taiwan; 4 Department of Information Management, National Taipei University of Nursing and Health Sciences, 365 Ming-Te Road, Beitou District, Taipei 11219, Taiwan; cyhsu@ntunhs.edu.tw; 5 Master Program in Global Health and Development, College of Public Health, Taipei Medical University, 250 Wu-Hsing Street, Taipei 11031, Taiwan; 6 School of Public Health, College of Public Health, Taipei Medical University, 250 Wu-Hsing Street, Taipei 11031, Taiwan; 7 Department of Computer Science and Information Engineering, Tamkang University, 151 Yingzhuan Road, Tamsui District, New Taipei 25137, Taiwan; billy72325@gmail.com; 8 National Health Research Institutes, 35 Keyan Road, Zhunan Town, Miaoli County 35053, Taiwan; 9 Department of Internal Medicine, Taipei Veterans General Hospital Yuanshan/Suao Branch, 301 Section 1, Subin Road, Suao Town, Yilan 27047, Taiwan; lmhsu@hotmail.com; 10 Department of Leisure Industry and Health Promotion, National Taipei University of Nursing and Health Sciences, 365 Ming-Te Road, Beitou District, Taipei 11219, Taiwan; pychang@ntunhs.edu.tw; 11 Research Center for Healthcare Industry Innovation, National Taipei University of Nursing and Health Sciences, 365 Ming-Te Road, Beitou District, Taipei 11219, Taiwan; 8lukas@ntunhs.edu.tw; 12 Nutrition Research Center, Taipei Medical University Hospital, 252 Wu-Hsing Street, Taipei 11031, Taiwan

**Keywords:** dietary patterns, factor analysis, serum hepatic enzyme, dyslipidemia, impaired fasting plasma glucose, cross-sectional study, Taiwan

## Abstract

We investigated the association between dietary patterns and serum hepatic enzyme levels in adults with dyslipidemia and impaired fasting glucose in Taiwan. A total of 15,005 subjects (5452 men and 9553 women) aged 35–69 years were selected. Two major dietary patterns were identified by principal component analysis: Western dietary pattern and Mediterranean dietary pattern. Subjects in the highest quartile (Q4) of the Western dietary pattern showed an increased risk of elevated serum alanine aminotransferase (ALT) levels (OR: 1.24, 95% CI: 1.06–1.45, *p*-trend = 0.01). Fur-thermore, in the highest quartile of the Western dietary pattern, subjects with high waist circum-ference were observed to have a greater risk for developing abnormal serum ALT levels compared to those in the lowest quartile (Q1) (OR: 1.43, 95% CI: 1.04–1.97, *p*-trend = 0.01). In the highest quartile of the Western dietary pattern, only women were at an increased risk for having abnormal serum ALT levels (OR: 1.28, 95% CI: 1.04–1.59, *p*-trend = 0.03). By contrast, in the highest quartile of the Mediterranean dietary pattern, only men were at a reduced risk for having abnormal serum gamma-glutamyl transferase (GGT) levels (OR: 0.72, 95% CI: 0.53–0.97, *p*-trend = 0.048). We report a positive association between the Western dietary pattern and abnormal serum ALT levels.

## 1. Introduction

Chronic liver disease is a major cause of morbidity and mortality and has become an increasing burden on the health care system [1]. Serum hepatic enzymes including alanine aminotransferase (ALT; glutamate pyruvate transaminase), aspartate aminotransferase (AST; glutamate oxaloacetate transaminase), and alkaline phosphatase (ALP) are used as markers for liver disease [1,2]. Metabolic syndrome, obesity, and nonalcoholic fatty liver disease (NAFLD) are disorders known to be associated with changes in serum enzymes [3]. Several conditions that injure the hepatic tissues may elevate serum levels of ALT and AST. One of the most common diseases that elevate liver function enzymes is NAFLD, which is described as a condition of fat accumulation in the liver in the absence of excessive alcohol intake [4,5]. However, the association between NAFLD and elevated serum transaminases is complex and not always linear. Indeed, many patients with mild NAFLD or chronic liver disease often had normal to near normal liver transaminases [1]. Routine laboratory test results should not be used as a sole screening tool to identify or exclude patients suspected of having chronic liver diseases [1].

Worldwide, NAFLD affects approximately 20–40% of the general population in Western countries and 5–40% in the general population across the Asia-Pacific region [5,6]. In Taiwan, the prevalence of NAFLD was 11.4–41% in two studies and the rates were higher in specific subgroups such as healthy taxi drivers and obese individuals from weight reduction programs [7,8]. With increasing trends in westernized lifestyle, such as inactive lifestyle and overnutrition, diabetes mellitus, overweight and obesity, as well as metabolic syndrome and NAFLD/nonalcoholic steatohepatitis are anticipated to be raised in Taiwan [8].

Besides overnutrition, further dietary factors are known to modify liver disease. For instance, enrichment of a hypocaloric diet with almonds was found to reduce body weight and improve serum ALT and AST levels [9]. Furthermore, the antioxidant content of fruits and vegetables [10], and the intake of lactic acid bacteria [11] have been proposed to have some impact on serum hepatic enzyme levels. Therefore, it is meaningful to investigate the association between dietary patterns and serum hepatic enzyme levels in a population-based cohort by conducting a dietary pattern analysis. To the best of our knowledge, no studies have investigated the association between dietary patterns and three serum hepatic enzyme levels including ALT, AST, and gamma-glutamyl transferase (GGT) in adults with borderline metabolic syndrome. Therefore, we investigated the association between dietary patterns and serum hepatic enzyme levels in Taiwanese adults aged 35–69 years with both dyslipidemia and impaired fasting plasma glucose.

## 2. Materials and Methods

### 2.1. Study Design

This study used a cross-sectional approach by collecting data from the Mei Jau (MJ) International Health Management Institution in Taiwan between 2001 and 2015. The MJ International Health Management Institution is an independent health screening and management institution in Taiwan including eight screening centers in Asia, with four of the centers located in major cities (Taipei, Taoyuan, Taichung, and Kaohsiung) in Taiwan. Upon the arrival at the health screening center, participants filled out a standard questionnaire regarding their demographics, medical history, diet, lifestyle, and exercise habits. A written consent form was also given to the participants, which allowed the MJ International Health Management Institution to collect data for research purposes only, and all personal information was kept confidential [12,13]. This study was approved by the Taiwan Medical University-Joint Institutional Review Board (N201810008).

### 2.2. Participants and Data Collection

Initially, the MJ database included 377,124 adults who visited the MJ Health Management Institution for health screening between 2001 and 2015. We retrieved data on subjects who were aged between 35 and 69 years and had both dyslipidemia and elevated fasting plasma glucose (FPG) (*n* = 20,266). This unique population as data from clinical and epidemiological studies had proven that NAFLD is a hepatic manifestation of metabolic syndrome [14,15]. We made an assumption that our selected subjects with borderline metabolic syndrome are at higher risk for having elevated serum hepatic enzyme levels. In terms of exclusion criteria, subjects with multiple entries in the MJ dataset, a family history of hyperlipidemia or diabetes, chronic diseases such as cancer, liver disorder, renal disorder, and diabetes mellitus, or were on thyroid, antibiotics, steroids, diabetic, or cardiovascular related medications (*n* = 174) were excluded. The term “multiple entries” refers to participants who had come to the MJ health screening center for health checkup more than once between 2001 and 2015. Subjects who were heavy drinkers (*n* = 313) or obese (*n* = 4246) were also excluded. Heavy drinkers were defined as consuming alcohol daily, and obese status was referred to body mass index (BMI) ≥ 27 kg/m^2^ [16]. After removing those with missing data (*n* = 528), a total of 15,005 subjects were included in the final analysis.

### 2.3. Definition of Dyslipidemia and Elevated Fasting Plasma Glucose

According to the National Cholesterol Education Program Adult Treatment Panel III guideline and the clinical cutoff value for abnormal lipid levels in Taiwan, dyslipidemia is defined as meeting one of the followings: (1) elevated triglycerides (TG) ≥ 2.3 mmol/L (200 mg/dL), (2) elevated total cholesterol (TC) ≥ 6.2 mmol/L (240 mg/dL), or (3) elevated low-density lipoprotein cholesterol (LDL-C) ≥ 4.1 mmol/L (160 mg/dL) [17]. However, in our study, we modified the criteria for dyslipidemia as having “borderline” lipid profile as the following: (1) elevated TG ≥ 1.7 mmol/L (150 mg/dL), (2) elevated TC ≥ 5.2 mmol/L (200 mg/dL), or (3) elevated LDL-C ≥ 3.4 mmol/L (130 mg/dL) [18]. Hyperglycemia, FPG ≥ 5.6 mmol/L (100 mg/dL) was defined as impaired blood glucose according to the guidelines set by the American Diabetes Association [19].

### 2.4. Definition of Abnormal Serum Hepatic Enzymes

Since serum hepatic enzymes such as ALT, AST, and GGT are tested routinely and automatically in most health screening centers and hospitals in Taiwan, our study applied this enzymological approach in evaluating liver function. Other diagnostic information related to liver injury such as presence of hepatotropic viruses, liver echogenicity, and liver biopsy was not available from the MJ database.

Abnormal serum hepatic enzymes in most clinical laboratories in Taiwan are referred to as ALT > 40 U/L, AST > 40 U/L, and GGT > 64 U/L [20,21]. However, we modified the cut-off points for elevated serum hepatic enzymes based on a preventive medicine perspective, and thus the cut-off points were stricter. In our study, ALT > 33 U/L, AST > 27 U/L, or GGT ≥ 50 U/L for men and GGT ≥ 39 U/L for women were considered as “abnormal” or “elevated” according to the reference ranges provided by the MJ laboratory [16]. The sex-specific cut-off points were used for GGT only in consideration of the variation on distributions of serum GGT levels by sex in our selected subjects from the MJ database [16].

### 2.5. Other Baseline Variables

A self-administered questionnaire was used to collect information on demographic characteristics, marital status, education, lifestyle habits, and menopausal status (women only). Subjects who responded as smoking on a daily basis, sometimes, or occasionally during the study period were classified as current smokers (yes), while the remainder were classified as non-smokers (no). Those who responded as drinking 1–2 times per week or more were classified as current drinkers (yes), while those who responded as none or drinking less than 1 time per week were classified as non-drinkers (no). Physical activity was classified as <150 min/week (sedentary) or ≥150 min/week (active) [12]. Anthropometric parameters such as weight, height, waist circumference, and BMI were measured during the health screening visit as mentioned in the previous study [22]. BMI was calculated by weight (kg) divided by the square of height (m^2^) and classified into three categories: underweight (BMI < 18.5 kg/m^2^), normal weight (18.5 ≤ BMI < 24 kg/m^2^), and overweight (24 ≤ BMI < 27 kg/m^2^) [23]. Abdominal obesity was defined as waist circumference >90 cm for men and >80 cm for women [24].

### 2.6. Blood Sampling and Laboratory Tests

All serum samples were analyzed at the central laboratory of the MJ Health Management Institution. Subjects were asked to fast overnight (12–14 h) prior to the examination date. Concentrations of TC, high-density lipoprotein cholesterol (HDL-C), TG, FPG, C-reactive protein (CRP), ALT, AST, and GGT were analyzed using commercial reagents [12], and LDL-C concentrations were calculated using the Friedewald’s formula [25].

### 2.7. Dietary Assessment

Dietary information was analyzed by using a standardized and validated semi-quantitative food frequency questionnaire (FFQ) developed by MJ Global Health Management [12,13,22]. The FFQ covered 22 food items commonly found in the Taiwanese eating pattern. The subjects were asked to answer how often they consumed each food item and portion during the past month (i.e., servings per day or per week from the lowest to the highest frequency) [12]. For example, the questions regarding the consumption of light- or dark-colored vegetables, vegetables with oil or dressing, or root crops contained five response options: <0.5 bowl/day, 0.5–1 bowl/day, 1–1.5 bowls/day, 1.5–2 bowls/day, and 2 bowls/day (1 bowl = 11 cm in diameter). For milk consumption, the five response options were: none or less than 1 glass/week, 1–3 glasses/week, 4–6 glasses/week, 1 glass/day, and 2 or more glasses/day (1 glass = 240 mL). For consumption of fruits and rice/flour products, five response options were: <1 serving/day, 1–2 servings/day, 2–3 servings/day, 3–4 servings/day, and ≥4 servings/day (1 serving = 1 medium sized apple, 1 bowl of rice, or 2 bowls of noodles). Lastly, for intake of other food items, the five response options were: <1 serving/week, 1–3 servings/week, 4–6 servings/week, 1 serving/day, and ≥2 servings/day. Meanwhile, each question also provided the definition and an example of one serving of the food consumed to ensure understanding of the question [12].

### 2.8. Statistical Analysis

Dietary patterns were identified by factor analysis. Orthogonal rotation with the Kaiser criterion was used to determine the number of factors (also known as dietary patterns) [26]. Food items were retained in the pattern if the absolute value of a factor loading was ≥0.25. Moreover, if the food item had the absolute value of a factor loading ≥0.25 in different dietary patterns, the dietary pattern was determined by the one which had a higher factor loading value. The two major factors (also known as dietary patterns) extracted by factor analysis were named according to the interpretation of existing data. Factor scores were calculated for each dietary pattern by summation of the consumption of food items weighed by their factor loadings. Within each dietary pattern, subjects were further divided into quartiles. Categorical variables are presented as a number or percentage, while continuous variables are presented as mean ± standard deviation (SD). For categorical data, the chi-square test was used to compare the differences in the characteristics of the subjects among quartiles of dietary patterns. For continuous variables, one-way analysis of variance (ANOVA) and Bonferroni post-hoc test were used for comparison. The linear regression analysis was performed in different multivariable adjusted models to examine the linear association between dietary patterns across quartiles and serum hepatic enzyme levels. The data were presented as regression coefficient (β) and 95% confidence interval (95% CI). To evaluate the associations between dietary patterns across quartiles and serum hepatic enzyme levels, a multivariable logistic regression analysis was performed, and the odds ratios (ORs) and 95% confidence intervals (CIs) were calculated. Two models were analyzed: model 1 was adjusted for gender, age, marital status, education, smoking, drinking, and physical activity, while model 2 was adjusted for variables included in model 1 plus BMI, waist circumference, abdominal obesity, components of dyslipidemia, FPG, and CRP. For the quartiles of each dietary pattern, the first quartile (Q1) was utilized as the reference group. In addition, we further performed two stratified analyses (stratification by waist circumference and gender) to assess the associations between dietary patterns across quartiles and serum hepatic enzyme levels. Statistical analyses in this study were conducted using SPSS 23 (IBM Corp., Armonk, NY, USA). All *p*-values presented are two-tailed, and *p* < 0.05 was considered significant.

## 3. Results

### 3.1. Dietary Patterns

Two major dietary patterns were identified by principal component analysis as shown in Table 1. The Western dietary pattern (pattern I) explained 13.97% of variance and consisted of eleven food items: eggs, meat, organ meats, refined bakery products, jam and honey, sugar-added beverages, rice/flour cooked in oil, deep-fried food, instant noodles, processed food, and sauces. The Mediterranean dietary pattern (pattern II) explained 12.78% of variance and consisted of eleven food items: milk, dairy products, legumes and soy products, seafood, dark-colored vegetables, light-colored vegetables, vegetables with oil or dressing, fruits, rice or flour products, whole grains, and root crops.

### 3.2. Characteristics of the Subjects and Dietary Patterns

The characteristics of subjects are shown in Table 2. There were 9553 (64%) women and 5452 (36%) men. Men had a higher proportion of having higher education (46.1% vs. 18.5%), being current smokers (28.6% vs. 3.1%), and current drinkers (23.9% vs. 5.4%) compared to women. Among women, 49.5% had experienced menopause and 50.5% still had regular menstruation. In terms of BMI and waist circumference, more men were overweight (52.9% vs. 29.1%), and men had higher waist circumference (82.3 ± 6.0 vs. 74.0 ± 6.2 cm) compared to women. Abdominal obesity was slightly more prevalent in women compared to men (18.6% vs. 10.5%). In terms of blood lipids and glucose, men had lower TC (5.63 ± 0.75 vs. 5.82 ± 0.77 mmol/L), HDL-C (1.32 ± 0.32 vs. 1.63 ± 0.41 mmol/L), LDL-C (3.55 ± 0.77 vs. 3.57 ± 0.76 mmol/L), and FPG (6.04 ± 1.05 vs. 6.08 ± 1.25 mmol/L) levels, but higher TG levels than women (1.76 ± 0.98 vs. 1.40 ± 0.75 mmol/L). However, men had lower CRP levels than women (0.21 ± 0.46 vs. 0.22 ± 0.41 mmol/L). Speaking of serum hepatic enzymes, men had a higher prevalence of having abnormal serum hepatic enzyme levels (ALT, AST, and GGT) compared to women, whereby the prevalence of abnormal serum ALT levels was the highest (34.7%).

The characteristics of subjects across quartiles of major dietary patterns are summarized in Table 3. In the highest quartile (Q4) of the Western dietary pattern, a higher proportion of younger age (35–44 years), being single, more educated, smoking, drinking, sedentary, non-menopausal, and overweight subjects were observed compared to the lower quartiles (Q1–Q3). Corresponding to BMI status, subjects in the highest quartile of the Western dietary pattern had higher waist circumference compared to those in the lower quartiles (Q1–Q3). Furthermore, subjects in the highest quartile of the Western dietary pattern had lower TC, HDL-C, and FPG levels compared to those in the lower quartiles (Q1–Q3). By contrast, subjects in the highest quartile of the Western dietary pattern had higher TG, ALT, and GGT levels compared to the lower quartiles (Q1–Q3).

In the highest quartile of the Mediterranean dietary pattern, a higher proportion of older age (55–64 years), being married, more educated, non-smoking, non-drinking, active physically, menopausal, and normal weight subjects were seen compared to the lower quartiles (Q1–Q3) (Table 3). Furthermore, subjects in the highest quartile of the Mediterranean dietary pattern had lower waist circumference, TC, LDL-C, TG, CRP, and GGT levels compared to those in the lower quartiles (Q1–Q3). Additionally, subjects in the highest quartile of the Mediterranean dietary pattern had higher HDL-C levels compared to those in the lower quartiles (Q1–Q3).

### 3.3. Association between Dietary Patterns and Serum Hepatic Enzymes

Table 4 presents the association between dietary patterns and serum hepatic enzyme levels in subjects. Our findings showed that adherence to the Western dietary pattern was positively associated with serum ALT and GGT levels in model 1. On the contrary, adherence to the Mediterranean dietary pattern was negatively associated with serum GGT levels in model 1. However, after fully adjusted for covariates in model 2, there was no significant association between the Western or Mediterranean dietary pattern and serum hepatic enzymes. Table 5 illustrates the odds ratios for abnormal serum hepatic enzyme levels across quartiles of dietary patterns. Subjects in the highest quartile of the Western dietary pattern had an increased odds of elevated serum ALT levels (OR: 1.38, 95% CI: 1.19–1.60, *p*-trend < 0.001) compared to those in the lowest quartile (Q1) of this dietary pattern in model 1. The result remained significant in model 2 (OR: 1.24, 95% CI: 1.06–1.45, *p*-trend = 0.011). However, no association was found between the Western dietary pattern and elevated serum AST or GGT levels in both models. Furthermore, there was no association between the Mediterranean dietary pattern and elevated serum hepatic enzyme levels in both models.

### 3.4. Stratification Analysis Results

As we further stratified the data by waist circumference (Table 6), our findings showed that in the highest quartile of the Western dietary pattern, subjects with high waist circumference were observed to have a greater risk for developing abnormal serum ALT levels compared to those in the lowest quartile (Q1) in model 2 (OR: 1.43, 95% CI: 1.04–1.97, *p*-trend = 0.01). Furthermore, in the highest quartile of the Mediterranean dietary pattern, subjects with high waist circumference were also observed to have a higher risk for developing abnormal serum ALT levels compared to those in the lowest quartile in model 2 (OR: 1.38, 95% CI: 1.00–1.90, *p*-trend = 0.04). However, as we further stratified the data by gender (Table 7), our findings indicated that, in the highest quartile of the Western dietary pattern, only female subjects had an increased risk for having abnormal serum ALT levels in model 2 (OR: 1.28, 95% CI: 1.04–1.59, *p*-trend = 0.03). Moreover, in the highest quartile of the Mediterranean dietary pattern, only male subjects were at a reduced risk for having abnormal serum GGT levels in model 2 (OR: 0.72, 95% CI: 0.53–0.97, *p*-trend = 0.048). However, there were no significant changes in odds ratios for abnormal serum hepatic enzyme levels across quartiles of the Western or Mediterranean dietary pattern in subjects with normal waist circumference compared to the reference group (Q1) in model 2.

## 4. Discussion

Our findings revealed that the Western dietary pattern was associated with an increased odds ratio of having abnormal serum ALT levels. On the contrary, the Mediterranean dietary pattern was negatively correlated with serum GGT levels in model 1. Furthermore, we stratified the subjects by waist circumference and gender. Our results showed that waist circumference and gender appeared to be modifiers for the association between dietary patterns and serum hepatic enzymes levels. Our work expands current knowledge on the association between diet and liver health in adults with “borderline” metabolic syndrome as well as the potential effects of waist circumference and gender on the association.

Elevated serum ALT, AST, and GGT levels have been proposed to be strongly related to cardiovascular disease (CVD), pre-diabetes, Type 2 diabetes, liver disease like NAFLD, metabolic syndrome, and some cancers [27]. Nutrition is considered to be a potential environmental factor influencing the risk of having abnormal serum hepatic enzyme levels [28]. The Western dietary pattern has long been known for its high content of saturated fats, proteins from red meat, sodium, and simple sugar like fructose and sucrose, and is low in fiber, vitamins, and minerals [29]. Our results showed that the Western dietary pattern was associated with an increased risk of having abnormal serum ALT levels. A previous study showed that a fast food diet was related to profound elevation in serum ALT levels among healthy participants [30]. Moreover, a limited number of studies have tried to explore the association between dietary patterns and serum hepatic enzymes, and the results suggested that a diet high in red meat, sweets, simple carbohydrates, and refined grains were positively associated with serum ALT and AST levels [31,32,33,34]. In clinical settings, mild elevation of serum hepatic enzymes in the upper normal range is often used as biomarker for NAFLD to avoid unnecessary diagnostic work-ups and enable early detection of NAFLD [35]. To put our findings within the context of these studies, the diet can accelerate the progression of liver injury even prior to appearance of clinical signs, which may be detectable by increased serum hepatic enzyme levels.

The Mediterranean diet has been known for its protecting effect against diabetes mellitus, metabolic syndrome, CVD, and NAFLD [36,37]. Overall, our findings showed that there were no significant changes in odds ratios for abnormal serum hepatic enzymes across quartiles of the Mediterranean dietary pattern. The Mediterranean dietary pattern had an inverse association with the risk of having abnormal serum GGT levels only in men. Similar to our findings, a cross-sectional study in Iranian adults did not find any significant association between their traditional dietary pattern (similar to the Mediterranean dietary pattern) and serum hepatic enzymes [28]. By contrast, fruit intake was inversely correlated with GGT levels, which might be due to β-carotene and vitamin C components [38]. Furthermore, vegetable intake was also negatively associated with serum ALT levels in Tehrani healthy adults in Iran [39]. In general, the Mediterranean dietary pattern is rich in antioxidants, phenols, fiber, vitamins, and minerals, and can prevent elevated serum hepatic enzyme levels through its protective effects against oxidative stress [38]. One possible explanation on why our study found no significant association between the Mediterranean dietary pattern and abnormal serum hepatic enzyme levels is that vegetables and fruits have contrasting effects compared to high-fat dairy products and sweets on common pathways of elevating serum hepatic enzymes, and their association with serum hepatic enzymes may interfered with each other [28].

Abdominal obesity, known as central obesity, was related to a variety of metabolic disorders and total mortality [40]. The most widely used measures for abdominal obesity include waist circumference and waist-to-hip ratio. In a few epidemiological studies, findings supported a role of abdominal adiposity independent from overall adiposity in predicting increased levels of serum hepatic enzymes [41,42,43]. Data from the Third National Health and Nutrition Examination Survey revealed that waist-to-hip ratio was more strongly associated with elevated serum ALT levels than BMI [43]. Our findings also observed that subjects with high waist circumference (also known as abdominal obesity) in the highest quartile of the Western dietary pattern had an increased risk for developing abnormal serum ALT levels. However, this relationship was not present in subjects with normal waist circumference. This subgroup finding is crucial for clinical application as obesity is a worldwide epidemic and linked to many chronic diseases [44,45]. Clinicians may use this finding to advocate for the importance of maintaining normal waist circumference in preventing obesity-associated liver disease.

At present, the influence of gender on common liver disease remains controversial. The association between dietary patterns and abnormal serum hepatic enzyme levels seems to be different between men and women. Our findings indicated that the Western dietary pattern appeared to be a risk factor for developing abnormal serum ALT levels for women but not for men. This may contribute to the fact that women lose estrogen protection after menopause. There are a number of physiological and biochemical changes in menopause, which can affect liver function and mediate the development of liver disease [46]. Another study also showed that sex hormones, particularly estrogen, appeared to play a role in the pathogenesis of NAFLD [47]. By contrast, the Mediterranean dietary pattern was found to offer some protective effects against abnormal serum GGT levels for men but not for women. This may also be explained by the estrogen deficiency and presence of abdominal obesity found in women, which weakened the beneficial effect of the Mediterranean dietary pattern against abnormal serum GGT levels. Moreover, men had higher serum GGT levels at baseline compared to women. Consequently, the aforementioned reasons may explain why the Mediterranean dietary pattern appears to be a protective factor against abnormal serum GGT levels for men only.

The study has several methodological limitations. First, the factor analysis was limited in terms of subjectivity in determining and naming dietary patterns. This may cause difficulties on extrapolating present findings to other populations. Second, there were more women than men (64% vs. 36%) recruited in our study, and thus the gender differences may be a result of such selection bias. Third, the MJ database lacked information more accurately reflecting liver function such as presence of hepatotropic viruses, liver echogenicity, and etiology of potential liver disease. Fourth, the exclusion of subjects with a family history of diabetes may result in an overestimation or underestimation of dietary effects on serum hepatic enzymes depending on gene-nutrition interactions. Despite these limitations, this study has several strengths. It is the first to investigate the association between dietary patterns and serum hepatic enzyme levels in adults with dyslipidemia and impaired fasting glucose, as well as one of the few studies to explore the correlations between dietary patterns and the three serum hepatic enzymes (ALT, AST, and GGT). Furthermore, this is a very large cohort study, and the use of the standardized and validated questionnaire also allowed more representative and consistent results for a similar population. Lastly, the key confounders related to serum hepatic enzymes were all controlled in this study.

## 5. Conclusions

This cross-sectional study addresses the association between dietary patterns and abnormal serum hepatic enzyme levels in adults with “borderline” metabolic syndrome in Taiwan. The Western dietary pattern was associated with a higher risk of having abnormal serum ALT levels. Abdominal obesity seemed to increase the risk of having abnormal serum ALT levels regardless of dietary patterns (Western or Mediterranean). Lastly, gender had some influence on the association between dietary patterns and abnormal serum hepatic enzyme levels. These findings have implications in terms of recommending patients with “borderline” metabolic syndrome to avoid the consumption of a Western dietary pattern, and adherence to a Mediterranean dietary pattern may provide protection against abnormal serum hepatic enzyme levels, particularly in men.

## Figures and Tables

**Table 1:** Factor loadings of two dietary patterns identified by principal component analysis ^1^.

Food Items	Pattern I Western Dietary Pattern	Pattern II Mediterranean Dietary Pattern
Milk	-	0.292
Dairy products	-	0.310
Eggs	0.439	-
Meat	0.523	-
Organ meats	0.488	-
Legumes/soy products	0.302	0.408
Seafood	-	0.374
Light-colored vegetables	-	0.764
Dark-colored vegetables	-	0.786
Fruits	-	0.558
Vegetables with oil/dressing	-	0.472
Rice/flour products	-	0.263
Whole grains	-	0.362
Root crops	-	0.501
Refined bakery products	0.378	-
Jam/honey	0.402	-
Sugar-added beverages	0.483	-
Rice/flour cooked in oil	0.483	-
Deep-fried food	0.646	-
Instant noodles	0.420	-
Processed food	0.586	-
Sauces	0.559	-

^1^ The absolute value of a factor loading ≥0.25 was used in the identification of dietary patterns. For simplicity, values <0.25 were excluded and indicated as symbol “-”.

**Table 2:** Characteristics of the subjects (*n* = 15,005) ^1^.

Variables	Total (*n* = 15,005)	Women (*n* = 9553)	Men (*n* = 5452)	*p*
Age (years), *n* (%)				<0.001
35–44	5363 (35.7)	2454 (25.7)	2909 (53.4)	
45–54	4666 (31.1)	3186 (33.4)	1480 (27.1)	
55–64	4018 (26.8)	3141 (32.9)	877 (16.1)	
65–69	958 (6.4)	772 (8.1)	186 (3.4)	
Marital status, *n* (%)				<0.001
Single	1546 (10.3)	953 (10.0)	593 (10.9)	
Married	11,794 (78.6)	7125 (74.6)	4669 (85.6)	
Divorced	1665 (11.1)	1475 (15.4)	190 (3.5)	
Education, *n* (%)				<0.001
<University	10,717 (71.4)	7781 (81.5)	2936 (53.9)	
≥University	4288 (28.6)	1772 (18.5)	2516 (46.1)	
Current smoking, *n* (%)				<0.001
No	13,155 (87.7)	9261 (96.9)	3894 (71.4)	
Yes	1850 (12.3)	292 (3.1)	1558 (28.6)	
Current drinking, *n* (%)				<0.001
No	13,190 (87.9)	9039 (94.6)	4151 (76.1)	
Yes	1815 (12.1)	514 (5.4)	1301 (23.9)	
Physical activity, *n* (%)				0.632
Sedentary	6185 (65.5)	4056 (65.3)	2129 (65.8)	
Active	3264 (34.5)	2157 (34.7)	1107 (34.2)	
Menopause, *n* (%)				-
No	4825 (50.5)	4825 (50.5)	-	
Yes	4728 (49.5)	4728 (49.5)	-	
BMI (kg/m^2^), *n* (%)				<0.001
<18.5 (underweight)	587 (3.9)	539 (5.6)	48 (0.9)	
18.5 ≤ BMI < 24 (normal)	8756 (58.4)	6236 (65.3)	2520 (46.2)	
24 ≤ BMI < 27 (overweight)	5662 (37.7)	2778 (29.1)	2884 (52.9)	
Waist circumference (cm)	77.0 ±7.3	74.0 ± 6.2	82.3 ± 6.0	<0.001
Abdominal obesity, *n* (%)				<0.001
No	12,651 (84.3)	7774 (81.4)	4877 (89.5)	
Yes	2354 (15.7)	1779 (18.6)	575 (10.5)	
TC (mmol/L)	5.75 ± 0.77	5.82 ± 0.77	5.63 ± 0.75	<0.001
HDL-C (mmol/L)	1.52 ± 0.41	1.63 ± 0.41	1.32 ± 0.32	<0.001
LDL-C (mmol/L)	3.56 ± 0.76	3.57 ± 0.76	3.55 ± 0.77	<0.001
TG (mmol/L)	1.53 ± 0.86	1.40 ± 0.75	1.76 ± 0.98	<0.001
FPG (mmol/L)	6.07 ± 1.18	6.08 ± 1.25	6.04 ± 1.05	0.024
CRP (mmol/L)	0.22 ± 0.43	0.22 ± 0.41	0.21 ± 0.46	<0.001
ALT (U/L)	27.9 ± 22.0	24.6 ± 20.7	33.7 ± 23.2	<0.001
normal, *n* (%)	11,694 (77.9)	8135 (85.2)	3559 (65.3)	
abnormal, *n* (%)	3311 (22.1)	1418 (14.8)	1893 (34.7)	
AST (U/L)	23.7± 11.1	23.0 ± 11.3	24.8 ± 10.7	<0.001
normal, *n* (%)	12,150 (77.2)	7984 (83.6)	4166 (76.4)	
abnormal, *n* (%)	2855 (19.0)	1569 (16.4)	1286 (23.6)	
GGT (U/L)	28.9 ± 33.5	24.5 ± 26.3	36.4 ± 42.4	<0.001
normal, *n* (%)	12,948 (86.3)	8400 (87.9)	4548 (83.4)	
abnormal, *n* (%)	2057 (13.7)	1153 (12.1)	904 (16.6)	

BMI: body mass index, TC: total cholesterol, HDL-C: high-density lipoprotein cholesterol, LDL-C: low-density lipoprotein cholesterol, TG: triglycerides, FPG: fasting plasma glucose, CRP: C-reactive protein, ALT: alanine aminotransferase, AST: aspartate aminotransferase, GGT: gamma-glutamyl transferase. ^1^ Data are presented as mean ± SD for continuous variables and *n* (%) for categorical variables. The Student’s *t* test and chi-square test were preformed to compare the continuous and categorical variables, respectively, between men and women. *n* = 9449 for physical activity data, *n* = 9553 for menopause data (women only).

**Table 3:** Characteristics of subjects across quartiles of major dietary patterns (*n* = 15,005) ^1^.

Variables	Western Dietary Pattern					Mediterranean Dietary Pattern				
	Q1 *n* = 3751	Q2 *n* = 3751	Q3 *n* = 3752	Q4 *n* = 3751	*p*	Q1 *n* = 3751	Q2 *n* = 3751	Q3 *n* = 3752	Q4 *n* = 3751	*p*
Factor score range	−2.51, −0.73	−0.73, −0.14	−0.14, 0.58	0.58, 5.75		−2.80, −064	−0.64, −0.12	−0.12, 0.55	0.55, 5.30	
Age (years), *n* (%)					<0.001					<0.001
35–44	637 (17.0)	1173 (31.3)	1526 (40.7)	2027 (54.0)		1548 (41.3)	1389 (37.0)	1303 (34.7)	1123 (29.9)	
45–54	1270 (33.9)	1254 (33.4)	1179 (31.4)	963 (25.7)		1174 (31.3)	1149 (30.6)	1176 (31.3)	1167 (31.1)	
55–64	1445 (38.5)	1076 (28.7)	875 (23.3)	622 (16.6)		833 (22.2)	986 (26.3)	1027 (27.4)	1172 (31.3)	
65–69	399 (10.6)	248 (6.6)	172 (4.6)	139 (3.7)		196 (5.2)	227 (6.1)	246 (6.6)	289 (7.7)	
Marital status, *n* (%)					<0.001					<0.001
Single	317 (8.5)	385 (10.3)	368 (9.8)	476 (12.7)		491 (13.1)	382 (10.2)	347 (9.3)	326 (8.6)	
Married	2892 (77.1)	2925 (78.0)	3020 (80.5)	2957 (78.8)		2803 (74.7)	2974 (79.3)	3003 (80.0)	3014 (80.4)	
Divorced	542 (14.4)	441 (11.7)	364 (9.7)	318 (8.5)		457 (12.2)	395 (10.5)	402 (10.7)	411 (11.0)	
Education, *n* (%)					<0.001					<0.001
<University	2994 (79.8)	2714 (72.4)	2544 (67.8)	2465 (65.7)		2797 (74.6)	2727 (72.7)	2602 (69.3)	2591 (69.1)	
≥University	757 (20.2)	1037 (27.6)	1208 (32.2)	1286 (34.3)		954(25.4)	1024 (27.3)	1150 (30.7)	1160 (30.9)	
Current smoking, *n* (%)					<0.001					<0.001
No	3537 (94.3)	3410 (90.9)	3256 (86.8)	2952 (78.7)		3090 (82.4)	3272 (87.2)	3355 (89.4)	3438 (91.7)	
Yes	214 (5.7)	341 (9.1)	496 (13.2)	799 (21.3)		661 (17.6)	479 (12.8)	397 (20.6)	313 (8.3)	
Current drinking, *n* (%)					<0.001					<0.001
No	3467 (92.4)	3374 (89.9)	3243 (86.4)	3106 (82.8)		3234 (86.2)	3266 (87.1)	3348 (89.2)	3342 (89.1)	
Yes	284 (7.6)	377 (10.1)	509 (13.6)	645 (17.2)		517 (13.8)	485 (12.9)	404 (10.8)	409(10.9)	
Physical activity, *n* (%)					<0.001					<0.001
Sedentary	1430 (58.0)	1565 (65.3)	1615 (68.2)	1575 (71.0)		1841(78.3)	1655 (69.5)	1434 (60.9)	1255 (53.1)	
Active	1034 (42.0)	833 (34.7)	754 (31.8)	644 (29.0)		510(21.7)	725 (30.5)	9 21(39.1)	1108 (46.9)	
Menopause, *n* (%)					<0.001					<0.001
No	1135 (38.8)	1274 (50.0)	1272 (56.7)	1144 (62.3)		1311 (56.5)	1213 (51.7)	1205 (50.2)	1096 (44.1)	
Yes	1787 (61.2)	1275 (50.0)	973(43.3)	693 (37.7)		1010 (43.5)	1132 (48.3)	1194 (49.8)	1392 (55.9)	
BMI (kg/m^2^), *n* (%)					<0.001					0.235
<18.5 (underweight)	173 (4.6)	159 (4.2)	132 (3.5)	123 (3.3)		152 (4.1)	143 (3.8)	150 (4.0)	142 (3.8)	
18.5 ≤ BMI < 24 (normal)	2372 (63.2)	2280 (60.8)	2165 (57.7)	1939 (51.7)		2181 (58.1)	2136 (57.0)	2189 (58.3)	2250 (60.0)	
24 ≤ BMI < 27 (overweight)	1206 (32.2)	1312 (35.0)	1455 (38.8)	1689 (45.0)		1418 (37.8)	1472 (39.2)	1413 (37.7)	1359 (36.2)	
Waist circumference (cm)	75.8 ± 6.9	76.4 ± 7.2	77.2 ± 7.4	78.6 ± 7.3	<0.001	77.3 ± 7.5	77.2 ±7.2	77.0 ± 7.2	76.7 ± 7.2	<0.001
Abdominal obesity, *n* (%)					0.024					0.29
No	3127 (83.4)	3200 (85.3)	3194 (85.1)	3130 (83.4)		3139 (83.7)	3168 (84.5)	3149 (83.9)	3195 (85.2)	
Yes	624 (16.6)	551 (14.7)	558 (14.9)	621 (16.6)		612 (16.3)	583 (15.5)	603 (16.1)	556 (14.8)	
TC (mmol/L)	5.80 ± 0.78	5.76 ± 0.76	5.76 ± 0.77	5.68 ± 0.77	<0.001	5.79 ± 080	5.75 ± 0.77	5.74 ± 0.76	5.73 ± 0.75	0.004
HDL-C (mmol/L)	1.57 ± 0.41	1.55 ± 0.42	1.51 ± 0.41	1.44 ± 0.38	<0.001	1.50 ± 0.41	1.51 ± 0.42	1.51 ± 0.40	1.54 ± 0.42	<0.001
LDL-C (mmol/L)	3.58 ± 0.76	3.55 ± 0.76	3.58 ± 0.76	3.54 ± 0.77	0.06	3.60 ± 0.80	3.55 ± 0.76	3.56 ± 0.75	3.54 ± 0.74	0.004
TG (mmol/L)	1.46 ± 0.79	1.51 ± 0.88	1.52 ± 0.78	1.62 ± 0.98	<0.001	1.57 ± 0.91	1.55 ± 0.90	1.52 ± 0.85	1.47 ± 0.79	<0.001
FPG (mmol/L)	6.11 ± 1.23	6.06 ± 1.12	6.06 ± 1.23	6.05 ± 1.10	0.040	6.08 ± 1.29	6.07 ± 1.16	6.06 ± 1.09	6.07 ± 1.16	0.72
CRP (mmol/L)	0.23 ± 0.55	0.21 ± 0.40	0.21 ± 0.39	0.21 ± 0.36	0.65	0.24 ± 0.56	0.22 ± 0.45	0.20 ± 0.26	0.21 ± 0.40	<0.001
ALT (U/L)	26.3 ± 22.1	27.4 ± 22.8	27.7 ± 21.8	30.1 ± 21.3	<0.001	28.3 ± 22.1	27.6 ± 21.6	28.3 ± 24.6	27.3 ± 19.7	0.20
AST (U/L)	23.9 ± 12.1	23.8 ± 12.0	23.3 ± 10.1	23.7 ± 10.2	0.12	23.6 ± 11.7	23.4 ± 10.8	24.0 ± 12.2	23.7 ± 9.6	0.36
GGT (U/L)	26.5 ± 39.1	28.2 ± 30.6	28.5 ± 28.4	32.2 ± 34.8	<0.001	31.1 ± 37.1	28.5 ± 28.4	28.6 ± 37.0	27.2 ± 30.6	<0.001

BMI: body mass index, TC: total cholesterol, HDL-C: high-density lipoprotein cholesterol, LDL-C: low-density lipoprotein cholesterol, TG: triglycerides, FPG: fasting plasma glucose, CRP: C-reactive protein, ALT: alanine aminotransferase, AST: aspartate aminotransferase, GGT: gamma-glutamyl transferase. ^1^ Data are presented as mean ± SD for continuous variables and *n* (%) for categorical variables. The *p*-values were derived from general linear regression for continuous variables and from chi-square test for categorical variables. *n* = 9449 for physical activity data, *n* = 9553 for menopause data (women only).

**Table 4:** Multivariable linear regression analysis of serum hepatic enzyme levels across quartiles of major dietary patterns (*n* = 15,005) ^1^.

Models of Dietary Patterns	Major Dietary Patterns				
	β (95% CI)				*p*
	Q1	Q2	Q3	Q4	
Serum ALT Level					
Western dietary pattern					
Model 1	Ref	0.74 (−0.50, 1.98)	0.38 (−0.87, 1.64)	2.32 (1.01, 3.63)	0.002 **
Model 2	Ref	0.64 (−0.57, 1.84)	−0.07 (−1.30, 1.15)	1.33 (0.05, 2.61)	0.13
Mediterranean dietary pattern					
Model 1	Ref	−1.01 (−2.26, 0.24)	0.20 (−1.07, 1.46)	−0.06 (−1.34, 1.21)	0.62
Model 2	Ref	−1.06 (−2.27, 0.16)	0.17 (−1.06, 1.40)	0.20 (−1.04, 1.44)	0.35
Serum AST Level					
Western dietary pattern					
Model 1	Ref	0.19 (−0.44, 0.83)	−0.37 (−1.02, 0.27)	0.33 (−0.34, 1.00)	0.75
Model 2	Ref	0.17 (−0.46, 0.80)	−0.50 (−1.13, 0.14)	0.07 (−0.60, 0.73)	0.63
Mediterranean dietary pattern					
Model 1	Ref	−0.68 (−1.31, −0.04)	−0.01 (−0.66, 0.63)	−0.23 (−0.88, 0.43)	0.995
Model 2	Ref	−0.67 (−1.30, −0.04)	−0.03 (−0.67, 0.61)	−0.13 (−0.77, 0.52)	0.80
Serum GGT Level					
Western dietary pattern					
Model 1	Ref	0.80 (−1.09, 2.67)	0.36 (−1.55, 2.26)	3.05 (1.05, 5.04)	0.008 **
Model 2	Ref	0.59 (−1.25, 2.44)	−0.26 (−2.13, 1.62)	1.98 (0.23, 3.95)	0.12
Mediterranean dietary pattern					
Model 1	Ref	−1.96 (−3.85, −0.06)	−1.52 (−3.44, 0.39)	−2.23 (−4.17, −0.29)	0.047 *
Model 2	Ref	−2.10 (−3.96, −0.24)	−1.55 (−3.43, 0.33)	−1.89 (−3.80, 0.01)	0.10

ALT: alanine aminotransferase, AST: aspartate aminotransferase, GGT: gamma-glutamyl transferase. ^1^ Data are presented as β and 95% confidence intervals (CI) in the parentheses. Model 1: adjusted for sex, age, marital status, education, smoking, drinking, and physical activity. Model 2: adjusted for variables in model 1 plus body mass index, waist circumference, components of dyslipidemia, and fasting plasma glucose. Among the quartiles, Q1 is the reference (Ref) group. * *p* < 0.05, ** *p* < 0.01.

**Table 5:** Odds ratios for abnormal serum hepatic enzyme levels across quartiles of major dietary patterns (*n* = 15,005) ^1^.

Models of Dietary Patterns	Major Dietary Patterns				*p*-Trend
	OR (95% CI)				
	Q1	Q2	Q3	Q4	
Abnormal Serum ALT					
Western dietary pattern					
Model 1	Ref	1.10 (0.95–1.28)	1.16 (1.00–1.35)	1.38 (1.19–1.60)	<0.001 ***
Model 2	Ref	1.10 (0.94–1.28)	1.10 (0.94–1.28)	1.24 (1.06–1.45)	0.011 *
Mediterranean dietary pattern					
Model 1	Ref	0.94 (0.82–1.09)	1.02 (0.88–1.17)	1.00 (0.86–1.16)	0.76
Model 2	Ref	0.93 (0.80–1.08)	1.02 (0.88–1.18)	1.04 (0.89–1.20)	0.44
Abnormal Serum AST					
Western dietary pattern					
Model 1	Ref	1.06 (0.91–1.23)	1.00 (0.86–1.16)	1.15 (0.99–1.34)	0.16
Model 2	Ref	1.05 (0.90–1.22)	0.96 (0.83–1.12)	1.08 (0.92–1.27)	0.56
Mediterranean dietary pattern					
Model 1	Ref	0.93 (0.80–1.08)	0.98 (0.85–1.14)	1.01 (0.87–1.17)	0.76
Model 2	Ref	0.93 (0.80–1.09)	0.99 (0.85–1.15)	1.04 (0.89–1.21)	0.49
Abnormal Serum GGT					
Western dietary pattern					
Model 1	Ref	1.04 (0.87–1.23)	0.95 (0.79–1.13)	1.14 (0.96–1.37)	0.29
Model 2	Ref	1.02 (0.86–1.22)	0.89 (0.74–1.07)	1.06 (0.88–1.27)	0.9
Mediterranean dietary pattern					
Model 1	Ref	0.81 (0.68–0.96)	0.879 (0.74–1.04)	0.88 (0.74–1.04)	0.24
Model 2	Ref	0.79 (0.67–0.95)	0.882 (0.74–1.05)	0.89 (0.75–1.07)	0.37

ALT: alanine aminotransferase, AST: aspartate aminotransferase, GGT: gamma-glutamyl transferase. ^1^ Data are presented as odds ratios (OR) and 95% confidence intervals (CI) in the parentheses. Model 1: adjusted for sex, age, marital status, education, smoking, drinking, and physical activity. Model 2: adjusted for variables in model 1 plus body mass index, waist circumference, components of dyslipidemia, and fasting plasma glucose. Among the quartiles, Q1 is the reference (Ref) group. * *p* < 0.05, *** *p* < 0.001.

**Table 6:** Odds ratios for abnormal serum hepatic enzyme levels across quartiles of major dietary patterns stratified by waist circumference (*n* = 15,005) ^1^.

Models of Dietary Patterns	Normal Waist Circumference (*n* = 12,651)					High Waist Circumference (*n* = 2354)				
	Q1	Q2	Q3	Q4	*p*-Trend	Q1	Q2	Q3	Q4	*p*-Trend
Abnormal Serum ALT										
Western dietary pattern										
Model 1	Ref	1.17	1.14	1.32	0.004 **	Ref	0.91	1.26	1.43	0.007 **
		(0.98–1.38)	(0.96–1.35)	(1.11–1.57)			(0.66–1.26)	(0.92–1.72)	(1.05–1.96)	
Model 2	Ref	1.15	1.07	1.18	0.15	Ref	0.92	1.223	1.43	0.01 **
		(0.97–1.37)	(0.90–1.28)	(0.99–1.41)			(0.66–1.27)	(0.89–1.69)	(1.04–1.97)	
Mediterranean dietary pattern										
Model 1	Ref	0.93	0.99	0.95	0.7	Ref	1.07	1.18	1.32	0.07
		(0.79–1.09)	(0.84–1.16)	(0.80–1.12)			(0.78–1.46)	(0.87–1.62)	(0.96–1.80)	
Model 2	Ref	0.88	0.96	0.96	0.88	Ref	1.1	1.24	1.38	0.04 *
		(0.75–1.04)	(0.81–1.14)	(0.81–1.14)			(0.79–1.5)	(0.90–1.71)	(1.00–1.90)	
Abnormal Serum AST										
Western dietary pattern										
Ref	1.11	0.95	1.09	0.75	Ref	0.88	1.17	1.24	0.09
		(0.94–1.31)	(0.79–1.13)			(0.91–1.30)	(0.68–1.13)	(0.85–1.61)		(0.90–1.71)
Model 2	Ref	1.09	0.92	1.04	0.8	Ref	0.89	1.13	1.25	0.1
		(0.92–1.28)	(0.77–1.09)	(0.87–1.24)			(0.64–1.23)	(0.82–1.57)	(0.90–1.73)	
Mediterranean dietary pattern										
Ref	0.89	0.93	0.97	0.89	Ref	1.13	1.23	1.2	0.23
		(0.75–1.06)	(0.79–1.11)			(0.82–1.15)	(0.82–1.56)	(0.89–1.70)		(0.87–1.66)
Model 2	Ref	0.88	0.92	0.99	0.94	Ref	1.14	1.26	1.22	0.18
		(0.74–1.04)	(0.78–1.10)	(0.83–1.18)			(0.82–1.59)	(0.91–1.75)	(0.88–1.70)	
Abnormal Serum GGT										
Western dietary pattern										
Model 1	Ref	1.06	0.89	1.12	0.61	Ref	1.01	1.12	1.07	0.6
		(0.87–1.29)	(0.73–1.10)	(0.93–1.38)			(0.68–1.42)	(0.78–1.60)	(0.74–1.53)	
Model 2	Ref	1.03	0.84	1.05	0.88	Ref	1	1.08	1.08	0.62
		(0.42–1.26)	(0.62–1.04)	(0.85–1.30)			(0.69–1.46)	(0.75–1.56)	(0.75–1.57)	
Mediterranean dietary pattern										
Model 1	Ref	0.77	0.88	0.84	0.19	Ref	1	0.9	1.09	0.8
		(0.63–0.93)	(0.73–1.07)	(0.68–1.02)			(0.70–1.43)	(0.63–1.29)	(0.73–1.56)	
Model 2	Ref	0.73	0.86	0.836	0.22	Ref	1.02	0.93	1.1	0.73
		(0.60–0.89)	(0.70–1.04)	(0.68–1.03)			(0.16–1.46)	(0.61–1.35)	(0.77–1.59)	

ALT: alanine aminotransferase, AST: aspartate aminotransferase, GGT: gamma-glutamyl transferase. ^1^ Data are presented as odds ratios and 95% confidence intervals in the parentheses. High waist circumference (≥80 cm for women and ≥90 cm for men) also refers to abdominal obesity. Model 1: adjusted for sex, age, marital status, education, smoking, drinking, and physical activity. Model 2: adjusted for variables in model 1 plus body mass index, components of dyslipidemia, and fasting plasma glucose. Among the quartiles, Q1 is the reference (Ref) group. * *p* < 0.05, ** *p* < 0.01.

**Table 7:** Odds ratios for abnormal serum hepatic enzyme levels across quartiles of major dietary patterns stratified by gender (*n* = 15,005) ^1^.

Models of Dietary Patterns	Men (*n* = 5452)					Women (*n* = 9553)				
	Q1	Q2	Q3	Q4	*p*-Trend	Q1	Q2	Q3	Q4	*p*-Trend
Abnormal Serum ALT										
Western dietary pattern										
Model 1	Ref	0.99	0.97	1.03	0.78	Ref	1.11	1.19	1.42	0.001 **
		(0.77–1.26)	(0.76–1.23)	(0.81–1.30)			(0.92–1.35)	(0.97–1.45)	(1.15–1.74)	
Model 2	Ref	0.95	0.93	0.96	0.82	Ref	1.12	1.14	1.28	0.03 *
		(0.74–1.24)	(0.72–1.19)	(0.75–1.24)			(0.92–1.36)	(0.93–1.40)	(1.04–1.59)	
Mediterranean dietary pattern										
Model 1	Ref	0.96	0.91	0.91	0.35	Ref	0.92	1.12	1.15	0.01 *
		(0.78–1.17)	(0.74–1.12)	(0.73–1.14)			(0.72–1.13)	(0.91–1.38)	(0.93–1.41)	
Model 2	Ref	0.93	0.95	0.94	0.64	Ref	1.04	0.92	1.1	0.07
		(0.75–1.16)	(0.76–1.18)	(0.75–1.19)			(0.80–1.35)	(0.71–1.20)	(0.86–1.42)	
Abnormal Serum AST										
Western dietary pattern										
Model 1	Ref	0.89	0.8	0.94	0.47	Ref	1.06	1.08	1.14	0.21
		(0.74–1.28)	(0.61–1.05)	(0.72–1.22)			(0.90–1.27)	(0.89–1.30)	(0.92–1.40)	
Model 2	Ref	0.94	0.77	0.91	0.4	Ref	1.07	1.06	1.07	0.5
		(0.71–1.23)	(0.59–1.01)	(0.70–1.19)			(0.89–1.18)	(0.87–1.28)	(0.87–1.32)	
Mediterranean dietary pattern										
Model 1	Ref	0.98	0.97	0.94	0.62	Ref	0.9	1	1.06	0.34
		(0.78–1.23)	(0.77–1.23)	(0.73–1.20)			(0.74–1.09)	(0.82–1.22)	(0.88–1.29)	
Model 2	Ref	0.97	0.99	0.97	0.86	Ref	0.91	1	1.09	0.24
		(0.77–1.23)	(0.78–1.26)	(0.76–1.25)			(0.71–1.11)	(0.82–1.22)	(0.90–1.33)	
Abnormal Serum GGT										
Western dietary pattern										
Model 1	Ref	1.1	1.03	1.14	0.53	Ref	1.01	0.9	1.13	0.61
		(0.79–1.54)	(0.75–1.42)	(0.83–1.56)			(0.82–1.24)	(0.72–1.12)	(0.90–1.42)	
Model 2	Ref	1.06	0.98	1.11	0.59	Ref	1.01	0.85	1.03	0.75
		(0.76–1.50)	(0.15–1.37)	(0.80–1.54)			(0.82–1.24)	(0.68–1.07)	(0.82–1.31)	
Mediterranean dietary pattern										
Model 1	Ref	0.81	0.89	0.7	0.043*	Ref	0.8	0.87	0.99	0.89
		(0.63–1.06)	(0.69–1.17)	(0.52–0.94)			(0.65–0.99)	(0.69–1.08)	(0.79–1.22)	
Model 2	Ref	0.79	0.87	0.72	0.048*	Ref	0.78	0.86	0.99	0.85
		(0.61–1.04)	(0.70–1.10)	(0.53–0.97)			(0.62–0.99)	(0.68–1.08)	(0.80–1.24)	

ALT: alanine aminotransferase, AST: aspartate aminotransferase, GGT: gamma-glutamyl transferase. ^1^ Data are presented as odds ratios and 95% confidence intervals in the parentheses. Model 1: adjusted for age, marital status, education, smoking, drinking, and physical activity. Model 2: adjusted for variables in model 1 plus body mass index, waist circumference, components of dyslipidemia, and fasting plasma glucose. Among the quartiles, Q1 is the reference (Ref) group. * *p* < 0.05, ** *p* < 0.01.

## Data Availability

The data that support the findings of this study are available from Mei Jau (MJ). Health Management Institute, but restricted for research use only. The data are not publicly available. Data are available from the authors upon reasonable request and with permission of MJ Health Management Institute.
